# Topics of Public Interest

**Published:** 1916-03

**Authors:** 


### TOPICS OF PUBLIC INTEREST.

**The Corning Hospital** has received a legacy of $5,000 from the estate of Mrs. George S. Sutherland.

**Investments.** The Medical World gives a list of 42 insurance companies which have gone out of existence. Most of these are for liability, accident, etc. Mention is also made of three fox fur companies capitalized, respectively at $31,250, $22,000 and $11,000 per pair of foxes owned. If you have money to invest, seek something substantial, that you can inspect and if necessary, manage yourself.

**Radium** has diminished in cost from $120,000 to $36,000 per grain, by the research of the U. S. Bureau of Mines.

**The Typhus Germ,** discovered by Harry Plotz of N. Y., has been verified during the epidemic in Serbia.

**The Nobel Prize,** 1914, has been awarded to Dr. Robert Barany of the University of Vienna for research in the physiology and pathology of the ear. No prize will be awarded for 1915, but an award will be made in 1916.

**Quarantine of Syphilis.** A woman of Springfield, Mo., has been forced to submit to quarantine by the Board of Health.

**Physicians Needed.** The Allies will shortly need 2,500 more English speaking doctors. Several well-paid vacancies exist in South Africa. Probably some of these positions will be open to Americans.

**The State Board of Health of Ohio** has ruled that the terms Dr., Doctor, M. D., Physician and Surgeon, may be used only by regularly graduated and licensed physicians.

**Health Insurance.** The American Association of Labor will introduce into the N. Y. legislature a bill providing for insurance of all men and women "gainfully employed" whose earnings are less than $1,200 a year. The bill is said to be modeled after German and British examples. For obvious reasons, such a bill should receive most careful attention from the medical profession. The general principle is good, but $1,200 is a rather high level of income at which to begin philanthropic intervention and, as has been shown by experience abroad, the details of such a bill may work injury to our profession.

**Medical Examination of Waiters.** Employees of hotels, restaurants and the like, engaged in preparing and serving food, are now required to be examined twice a year in Toronto. A good idea. We happen to know of a favorite soda fountain that has recently been avoided by several intelligent laymen, or at least laygirls, because the dispenser was pimply. So far as an ambulance diagnosis is dependable, he probably has nothing worse than acne and not the terrible infliction as to which the children are now being educated, but the effect on business is the same.

---

**The Millionth Model T Ford Motor** was completed at 1:53:30 p. m., Dec. 10, 1915. It is said to be exactly the same as the first of this model, not of the Ford in general, turned out in October, 1908. This gives one an idea of the rapid automobilization of the country.

---

**Compound Interest.** In judging of the value of an investment, the following table compiled by the Boston News Bureau is useful. It assumes that $100 is deposited on the first of each year and that interest and principal are not touched. It is practically correct only for the first two columns, representing bank interest, since it is impossible to compound interest immediately in ordinary investments. Allowance would also have to be made for the transfer of a part of the sum, whenever the total approached the limit allowed by a savings bank:

| At end of year: | 3% | 4% | 5% | 6% |
|---|---|---|---|---|
| 1 | $103 | $104 | $105 | $106 |
| 2 | 209 | 212 | 215 | 218 |
| 3 | 318 | 325 | 331 | 337 |
| 4 | 431 | 442 | 453 | 464 |
| 5 | 547 | 563 | 580 | 598 |
| 6 | 666 | 690 | 714 | 739 |
| 7 | 789 | 821 | 855 | 890 |
| 8 | 916 | 958 | 1,003 | 1,049 |
| 9 | 1,046 | 1,101 | 1,158 | 1,218 |
| 10 | 1,181 | 1,249 | 1,321 | 1,397 |
| 11 | 1,319 | 1,403 | 1,492 | 1,587 |
| 12 | 1,462 | 1,563 | 1,671 | 1,788 |
| 13 | 1,609 | 1,729 | 1,860 | 2,002 |
| 14 | 1,760 | 1,902 | 2,058 | 2,228 |
| 15 | 1,916 | 2,082 | 2,266 | 2,467 |
| 16 | 2,076 | 2,270 | 2,484 | 2,721 |
| 17 | 2,241 | 2,465 | 2,713 | 2,991 |
| 18 | 2,412 | 2,667 | 2,954 | 3,276 |
| 19 | 2,587 | 2,878 | 3,207 | 3,579 |
| 20 | 2,768 | 3,096 | 3,472 | 3,899 |

From a calculation which we made some time ago, with regard to life insurance, we are inclined that the above table refers to compounding once a year, whereas some banks compound every quarter and pay 4%. For instance, here are the figures on a 20-year policy, represented as being a good investment aside from the insurance value. Except that the actual amounts were reduced to the basis per $1,000 insurance, they represent an actual experience. The total amounts differ somewhat from those of the table as premiums were paid semi-annually and allowance was made for compounding interest at the same intervals:

| | |
|---|---|
| Total premiums paid | $600.01 |
| Surrender value at end of 20 years | 626.53 |
| Actual interest on investment | 26.52 |
| Value of accumulated premiums at 4% simple | 849.12 |
| Value of premiums at 4% compounded semi-annually | 929.58 |
| Loss as compared with savings bank | 303.05 |

It should be conceded that, according to actuary's statistics, the actual average payment for death losses, with interest, would come to about $206 on any single policy of $1,000. Thus, as compared with the savings bank, the cost of the insurance factor was about $97. At 5% compound interest, which can easily be made on conservative investments, the total value of the premiums should have reached about $1,042. The loss on the insurance investment, on this basis would be about $415, and the charge made by the company for skill in investing, clerical labor, etc., would be about $210 beyond the average loss in paying death claims. It will be noted that the premium paid on $1,000 insurance was almost exactly $30 a year but, owing to a temporary excess charge and subsequent rebate on passing a suppositious danger age, this average was not exactly maintained. It should be distinctly understood that no disparagement of life insurance is intended but, so far as we can gather from various reports, insurance has no value as an investment aside from the insurance itself. On the contrary, one sacrifices just about half of the actual cash invested, as compared with a savings bank deposit.

---

**The Commission on Cancer** of the Medical Society of the State of Pa., reports that the campaign was assisted by the co-operation of 66 medical journals (including this), that 38 counties in Pennsylvania held special meetings devoted to the consideration of cancer, with a total attendance of 1419, 20% of the total membership of the state society. Nothing is said as to actual accomplishments toward the solution of the cancer problem. Cancer deaths (in Pennsylvania) increased from 60.7 per 100,000 population in 1906 to 66.4 in 1911. Note: This increase may not be reliable. Estimates of population are apt to increase with undue rapidity in the years between censuses and to be suddenly set back to actual facts when the census is published. Obviously if this occurs, an increase in death rate from any cause would be noted. Again, we must allow for improvements in diagnosis, especially in regard to the detection of internal cancers, and for greater and greater efforts to secure full death reports, especially from cancer. Meantime, the average age of the population has been steadily increasing on account of sanitary and other improvements, so that more cancer material is available.

---

**Diabetic Foods.** The U. S. Dept. of Agriculture has ruled that ground gluten, gluten flour and self-raising gluten flour must contain not over 10% of water; not less than 14.2% of nitrogen in the water-free ground gluten, nor less than 7.1% in the water-free flour; ground gluten must contain not over 15% of nitrogen-free extract (protein factor 5.7); gluten flour not over 56%; ground gluten must contain not more than 5.5% of starch, gluten flour not more than 44% (diastatic method). A food designated as "diabetic" must contain not more than the proportion of glycogenic carbohydrates as the normal food of the same class, and the label must not give the impression that it may be used in unlimited quantities.

---

**Change in City Physicians for Buffalo.** The council has reported in favor of substituting four physicians at $1,800 for the 10 at various low salaries. Four health stations will be provided and the physicians will be on duty from 8.30 till 2, and will serve at dispensaries from 2 till 4. In addition to their regular duties, they will examine tuberculous suspects, care for sick school children and look after the milk stations. If the city physicians are still expected to respond to emrgency calls and attend obstetric cases at night, they will earn their salaries.

---

**Administration of Anesthetics.** The New York Society of Anesthetists has notified the County Medical Societies of New York State, that it proposes to introduce an amendment to the Public Health Law (Art. VIII, Sec. 160, par 7) to make it illegal for anyone except a licensed physician to administer a general anesthetic.

At first thought this would seem right and proper, as this change is apparently for the purpose of preventing unauthorized persons from practicing medicine. But upon careful consideration it is found to be a very radical step which would work great inconvenience and damage upon surgeons and obstetricians, and great injustice upon the nursing profession. A law making it illegal for anyone to give an anesthetic except as **prescribed** by a regularly licensed physician or surgeon would be much better.—Crouse-Irving Bull. Jan.

---

**Sanatarium Scandal.** A committee induced the Onondaga County Board of Supervisors to provide a hospital for 125 tuberculosis patients at a cost of about $175,000. A hospital is being completed at a cost of nearly $600,000 and a capacity of about 80 patients. Apparently about $42,000 of the $600,-000 has been actually stolen, and public opinion is boiling with wrath to punish the petty thieves who divided this spoil. The fact that nearly $400,000 was wasted does not seem to cause much anxiety.—Crouse-Irving Bull., Jan.

---

**Members of Legislature, Western N. Y.** Senate:

38. J. H. Walters, 315½ Genesee Street, Syracuse.

39. W. H. Hill, Lestershire.

40. C. J. Hewitt, R., Locke.

41. M. S. Halliday, R., Ithaca.

42. T. B. Wilson, R., Hall.

43. C. D. Newton, R., Geneseo.

44. A. D. Sanders, R., Stafford.

45. G. F. Argetsinger, R., Rochester.

46. J. B. Mullan, R., Rochester.

47. G. F. Thompson, R., Middleport.

48. C. T. Horton, R., 905 D. S. Morgan Bldg., Buffalo.

49. S. J. Ramsperger, D., 232 Emslie Street, Buffalo.

50. W. P. Greiner, D., 1037 Walden Avenue, Buffalo.

51. G. E. Spring, R., Franklinville.

Assembly:

Allegany—W. Duke, Jr., R., Wellsville.

Cattaraugus—D. H. Ames, R., Franklinville.

Cayuga—W. F. Whitman, R., Venice Center.

Chautauqua—L. L. Fancher, R., Jamestown; J. A. McGinnies, R., Ripley.

Chemung—R. P. Bush, D., Horseheads.

Chenango—B. Lord, R., Afton.

Erie—1. Alex. Taylor, R., 115 Franklin Street, Buffalo; 2. R. Graves, 68 Manchester Place, Buffalo; 3. N. J. Miller, R., 12 Cayuga Street, Buffalo; J. M. Mead, D., 137 Gold St., Buffalo; 5. J. A. Lynch, D., 694 So. Division Street, Buffalo; P. C. Jezewski, R., 173 Stanislaus Street, Buffalo; 7. J. J. Rolmhild, R., 31 Burch Avenue, Buffalo; 8. L. W. Gibbs, 15 Depew Avenue, Buffalo; 9. W. W. Cheney, R., Eden.

Genesee—L. H. Wells, R., Pavilion.

Livingston—G. F. Wheelock, R., Leicester.

Madison—M. E. Tallett, R., DeRuyter.

Monroe—1. J. A. Harris, R., Penfield; 2. S. L. Adler, R., Rochester; 3. J. R. Powers, R., Rochester; 4. F. Dobson, R., Charlotte; 5. F. W. Judson, R., Gates.
Niagara—1. W. Bewley, R., Lockport; 2. A. N. Parker, R., Niagara Falls.
Oneida—1. J. J. Hess, D., Utica; 2. L. M. Martin, R., Clinton; 3. G. T. Davis, R., Rome.
Onondaga—1. E. A. Arnts, R., 628 No. Noseti Street, Syracuse; 2. J. L. Kincaid, R., Robineau Road, Syracuse; 3. Geo. Fearon, R., 511 Van Buren Street, Syracuse.
Ontario—H. E. Wheeler, R., East Bloomfield.
Orleans—A. A. Comstock, R., Waterport.
Schuyler—H. J. Mitchell, R., Watkins.
Seneca—W. J. Maher, R., Seneca Falls.
Steuben—1. R. B. Oldfield, R., Bath; 2. R. M. Prangen, R., Hornell.
Wayne—R. A. Wilson, R., Savannah.
Wyoming—J. Knight, R., Arcade.
Yates—H. S. Fullager, R., Penn Yan.

---

**The Charity Eye, Ear and Throat Hospital of Erie County.** located at the corner of Broadway and Michigan Avenue, Buffalo, has recently issued its 24th Annual Report, for the year ending Sept. 30., 1915. 3,261 new patients were treated and 346 old patients. 3,875 different diseased conditions were noted. A total of 12,659 treatments were given and 1,076 operations were performed. This institution is supported partly by an appropriation by the Supervisors, partly by donations. It is a most worthy charity. The staff is as follows:

Eye department—Surgeons in charge, Benjamin H. Grove, M .D., John J. Finerty, M. D., John D. Flagg, M. D.; Associate Surgeons, H. H. Glosser, M. D., Edmond E. Blaauw, M. D., Herman D. Andrews, M. D.; Assistant Surgeons, A. F. Luhr, M. D., Robert S. Taylor, M. D., W. C. Lewin, M. D., Clara A, March, M. D.; Clinical Assistant, Joseph C. O'Gorman, M. D.

Ear, Nose and Throat department—Surgeons in charge, George F. Cott, M. D., E. A. Forsyth, M. D., Clayton M. Brown, M. D.; Associate Surgeons, Wm. F. Jacobs, M. D., George R. Turk, M D., Walter J. M. Wurtz, M. D., Thos. W. Connors, M. D., R. C. Conklin, M. D., Chester C. Cott, M. D., Frank C. Kleckner, M. D., Harry N. Feltes, M. D.; Assistant Surgeons, Francis A. Drake, M. D., Royal A. Paxton, M. D., Charles B. Handel, M. D.

Nervous department—Edward A. Sharp, M. D., physician in charge.

Consultants—Surgeons, Edward J. Meyer, M. D., Marshall Clinton, M. D.; Physicians, Henry C. Buswell, M. D., Earl P. Lothrop, M. D.; Neurologist, James W. Putnam, M. D., Edward A. Sharp, M. D.; X-Rayist, Leonard Reu, M. D.; Electro-

Therapeutics, A. W. Bayliss, M. D.; Dermatologist, Grover W. Wende, M. D.; Pathologists, Charles A. Bentz, M. D., William F. Jacobs, M. D.; Nurse in charge, Miss Catherine Zimmel, R. N.; Registrar, Miss Tillie Mayer.

---

**The Research Institute of the National Dental Association** was incorporated June 24, 1915. It occupies two large residences on Euclid Avenue, Cleveland. Its first report includes lists of directors, members, etc., and of problems begun or contemplated. Among these are systemic diseases resulting from oral infections, such as various joint diseases, iritis, nephritis, gastric and duodenal ulcer, glandular infections, pneumonia, pyorrhoea, dental caries, the brown stain of teeth, whose cause is entirely unknown, a platinum substitute (dentists using one-third of the world's production, valued at 2½ millions), filling of infected roots, oral bacteriology, salivary disorders, facial deformities, relation of ductless glands to dentistry, orthodontia, are included. Special research rooms will be available for visiting dentists. If this last item means that facilities will be offered to the profession generally, without special influence or appointment, it is not only of value in itself but as a precedent to similar medical institutions.

---

**Choice of Specialties at Harvard Medical School.**

| | 1st Year Choice | 4th Year Choice | Unchanged | Changed |
|---|---|---|---|---|
| Surgery | 17 | 16 | 11 | 6 |
| Internal medicine | 5 | 10 | 4 | 1 |
| General practice | 10 | 10 | 6 | 4 |
| Pediatrics | 5 | 9 | 4 | 1 |
| Gynecology and obstetrics | 4 | 6 | 2 | 2 |
| Public health | 1 | 3 | 1 | 0 |
| Eye and ear | 2 | 2 | 2 | 0 |
| Orthopedics | 4 | 1 | 1 | 3 |
| Neurology | 7 | 1 | 0 | 7 |
| Genito-Urinary | 0 | 1 | 0 | 0 |
| Nose and throat | 1 | 1 | 1 | 0 |
| Physiology | 0 | 1 | 0 | 0 |
| Pathology and bacteriology | 2 | 0 | 0 | 2 |
| X-ray | 1 | 0 | 0 | 1 |
| Uncertain | 11 | 5 | 1 | |

—Alumni Assn. Bulletin.

---

**Range of Ceanothus Sanguineus.** This has been considered limited to the western slope of the Rocky Mountains or, at most to the headwaters of the Missouri. Oliver A. Farwell of the Botanic Dept. of Parke, Davis & Co., reports in Rhodora, vol. 17, 1915, its discovery in the Keweenaw Peninsula, Mich., as early as 1886, when it was thought to be C. Americanus, and its recent identification as C. Sanguineus. (Note: C. Americanus contains about 9% of tannin and was used as an astringent, locally and internally. We wish to emphasize, not so much the value of such field work as a basis for pharmacology as the general availability of botany as a hobby for physicians, especially since the advent of the automobile. There is opportunity for new discovery along these lines, even in our own territory, not to mention the possibility of collecting medicinal herbs of considerable pharmacologic and commercial value. But we admit that, personally, no binomial Latin plant that grows is so interesting as a bare field that may yield Indian relics.).

**Vital Statistics of Buffalo.**

| | 1882 | 1891 | 1910 | 1913 | 1914 | 1915 |
|---|---|---|---|---|---|---|
| Population | 175,000 | 255,664 | 423,715 | 446,889 | 454,112 | 461,887 |
| Total deaths—all ages | 4,212 | 6,001 | 6,940 | 7,043 | 7,040 | 6,853 |
| General death rate | 24.06 | 23.47 | 16.37 | 15.76 | 15.50 | 14.83 |
| Living births | 4,809 | 8,309 | 10,008 | 11,867 | 12,612 | 12.683 |
| Birth rate | 27.47 | 32.49 | 23.61 | 26.55 | 27.77 | 27.45 |
| Deaths under one year | 1,110 | 1.786 | 1,641 | 1,631 | 1,533 | 1.373 |
| Deaths under one year during three summer months | 509 | 678 | 516 | 584 | 494 | 370 |
| Deaths under five years | 2,043 | 2,609 | 2,323 | 2,070 | 1,936 | 1,829 |
| Premature births | 44 | 145 | 261 | 295 | 306 | 290 |
| Stillbirths | 221 | 398 | 393 | 534 | 545 | 491 |

Note—Premature births are counted as births and deaths; stillbirths are not included in births nor deaths, but counted separately.

The years in the above table are selected for the following reasons: 1882 was the first year when accurate records were kept; in 1891 the new city charter, employing a full-time health commissioner, went into effect; in 1910 the present health commissioner was appointed, while the last three years are for immediate comparison.—Buffalo Sanitary Bulletin.

We would like to publish similar statistics for other towns in Western New York.

**University Day.** In accordance with precedent, the University of Buffalo celebrated Washington's Birthday. The Nu Sigma Nu, I. C. I. Chapter, held a dinner dance the evening before. The formal exercises of the University were held at 11 a. m., Feb. 22, with an address by George W. Wickersham, formerly Attorney General, and a historic review by Rev. A. V. V. Raymond, D. D., LL. D., of the movement to secure an Arts Dept. for the University, beginning with the election of Charles P. Norton, as Vice Chancellor in 1909. Rumors that have been current for some time were confirmed by the announcement of gifts of $500,000 from the family of the late Seymour H. Knox, which rendered possible the formal transfer of the building of the Women's Educational and Industrial Union on Niagara Square. Mrs. George W. Townsend, the founder, for many years President and of late years Honorary President of this institution, made a brief address. Gen. Edmund Hayes also gives $250,000, conditional on the raising of a total of $1,000,000. The Arts Dept. is already giving a two years' course, is thus expected to be in full operation in 1919.

The second annual dinner of the Associated Alumni was held at the Statler in the evening, Dr. Thos. H. McKee, Dean of the Medical Dept., acting as toastmaster.

The U. of B. Dramatic Club presented A Night Off at the Majestic Theatre on the evenings of Feb. 25 and 26.

---

**Membership Roster of State Medical Societies.** Percentage of members to total number of physicians in the state as per 1915 report of A. M. A.

| | | | | | |
|---|---|---|---|---|---|
| Hawaii | .75 | N. Dak. | .54 | Utah | .48 |
| Wis. | .73 | Georgia | .54 | California | .47 |
| Virginia | .70 | R. I. | .54 | Kansas | .47 |
| N. H. | .70 | Wash. | .54 | So. Dakota | .47 |
| Alabama | .69 | Penn. | .53 | Porto Rico | .47 |
| Minn. | .66 | New Jersey | .53 | Oregon | .45 |
| Illinois | .63 | Maine | .52 | Florida | .45 |
| N. Car. | .61 | Maryland | .52 | Arkansas | .44 |
| Mass. | .60 | New York | .52 | Delaware | .41 |
| Mich. | .60 | Ohio | .50 | Louisiana | .40 |
| Vermont | .57 | Kentucky | .50 | Dist. of Col. | .39 |
| Arizona | .57 | W. Va. | .50 | Colorado | .38 |
| Conn. | .57 | Wyoming | .50 | New Mex. | .34 |
| Texas | .57 | Indiana | .49 | Tennessee | .34 |
| Neb. | .56 | Missouri | .49 | Nevada | .32 |
| Canal Zone | .55 | Oklahoma | .49 | Idaho | .31 |
| Iowa | .55 | Montana | .49 | Phil. Islands | .13 |
| S. Car. | .55 | Mississippi | .48 | | |

---

The British War Office reports the **Mortality of Troops** to be 1:3 wounded; in the trenches, 1:2 wounded. We understand that this excludes strictly medical mortality. For previous wars, the ratio of killed (including deaths shortly after receiving wounds) to wounded has been about 1:4 or 5.

---

**Registration Area Mortality 1914.** This area now comprises about 2-3 of the total population of the U. S. 898,059 deaths were reported, about 13.5:1000. More than 30% of all deaths were due to three causes, heart disease, tuberculosis and pneumonia; and more than 60% were due to 11 causes, including also nephritis, cancer, diarrhoea and enteritis, apoplexy, arterial diseases, diphtheria, diabetes and typhoid.. Tuberculosis was the cause of 96,903 deaths, of which 84,366 were pulmonary or acute miliary. It will be noted that the long-standing rule that 1-10 of all deaths are due to tuberculosis still holds approximately true. But it is also true that deaths from tuberculosis have declined at almost exactly the same rate as the general mortality. Prior to 1904, the tuberculosis death rate fluctuated close to a rate of 2:1000 population, although even at that date, the rate of 20:1000 for all deaths in relation to population, could not longer be considered normal. Since then, there has been a pretty steady decline to 1.468:1000 for tuberculosis. Pneumonia gave a death rate of 1.27:000, the lowest on record (Total deaths 83,804). The only other group of diseases giving a death rate of more than 1:1000 poulation, was Bright's disease and nephritis, total 67,545 deaths, 1.024: 1000.

---

**Narcotic Law Notes.** The press continues to relate convictions for non-payment of license fees, charges of smuggling drugs, voluntary presentation of victims for treatment, etc. While we have not been in entire sympathy with the discrepancies between national and state laws and between rulings and actual facts, we must confess that these laws have exercised a marked influence on narcotic addiction, although they have also shown that the evil had been exaggerated.

---

**The Emergency Hospital** of Buffalo, by a minstrel show given by The French Club, and by subscriptions, has raised a fund for the equipment of the X-ray laboratory.

---

**The New York Committee for the Prevention of Blindness** was organized about seven years ago, under the auspices, and with the financial assistance of the Russell Sage Foundation. It owes its origin largely to the efforts of the Chairman of the Committee, Miss Louise Lee Schuyler, who is also a member of the Board of Directors of the Russell Sage Endowment Fund.

The report of the State Commission for the Blind had graphically shown that a large amount of the existing blindness might have been prevented had proper measures been taken, and emphasized the necessity of an organized movement to protect the eyes wherever they were in danger from whatever cause. It was found that about one quarter of all blindness in state schools for the blind everywhere was due to neglect of proper protective measures of infants at birth. The educational measures which have been so largely inaugurated by the American Medical Association, and carried out by State Boards of Health, have already materially reduced the number of victims that annually were due to infections of this character.

The State National Committee, with all of its machinery for carrying on an effective campaign, soon found that it was being overwhelmed by demands for advice and assistance in the propaganda for the conservation of sight from other states, so last year additional support was obtained from the John D. Rockafeller Foundation, a very generous contribution made by the citizens of Buffalo, and the scope of the Committee made national.

Special efforts have been made on the line of public education and of the enactment and enforcement of such laws as may be necessary to prevent unnecessary blindness or the injurious use of the eyes. The special lines to which attention has been directed has been that of opthalmia neonatorum through health departments and otherwise, legal enactments against the sale of improperly labeled wood alcohol, with advice to the public as to its toxic effect, especially in causing atrophy of the optic nerves, in securing proper lighting in schools, public buildings and homes, and in preventing needless accidents in workshops and elsewhere, which is a very prolific cause of loss of sight, and in making more widespread of the existence, the danger and the control of trachoma. A popular lecture has been provided and a series of lantern slides prepared which will be loaned to public speakers as a method of popular education on this subject. These slides, with the synopsis of the lecture, will be loaned without charge, the borrower paying only for transportation and any breakage which may occur.

A field agent, in the person of Mr. Gordon L. Berry, has been employed and he is now occupied in visiting many of the southern states in the development of the propaganda for the prevention of blindness. The work which he has already accomplished has been of great value. He is working in co-operation with the medical associations, the boards of health, local oculists, church societies and others interested in public welfare, and has already developed great enthusiasm for the work in the districts which he has visited. A large number of valuable publications have been issued by the committee which are distributed gratuitously or at cost.

It was proposed that the secretary would shortly visit Buffalo for the purpose of developing in conjunction with the Health Department, the Department of Public Education, the medical profession and others, a careful study as to the prevalent causes of blindness as they were found there, with the purpose of making that city a basis for preventive measures which could be used elsewhere. The resignation of the secretary,

Miss Van Blarcom, who has accepted the position of secretary for the Illinois Society for the Conservation of Vision in Chicago, while it will greatly broaden the work, will at the same time delay for a time the more intensive work which is in prospect. It is evident, however, that a movement is now well under way which gives promise of being of incalculable benefit to every community which it reaches. Reports of this work will appear in the Journal from time to time.—F. Park Lewis.

---

